# Advances in Artificial Intelligence for Non-ECG-Gated Coronary Artery Calcium Scoring: A Scoping Review

**DOI:** 10.7759/cureus.94019

**Published:** 2025-10-07

**Authors:** Francis E O'Toole, Maryam Zaffer, Jessica Cohen, Mathew Allard, Chase M Kingsbury, Rohit Muralidhar, Robin J Jacobs

**Affiliations:** 1 Medicine, Dr. Kiran C. Patel College of Osteopathic Medicine, Nova Southeastern University, Fort Lauderdale, USA; 2 Medical Education, Dr. Kiran C. Patel College of Osteopathic Medicine, Nova Southeastern University, Fort Lauderdale, USA

**Keywords:** artificial intelligence (ai), cardiovascular risk stratification, coronary artery calcium (cac) scoring, deep learning (dl), machine learning (ml), non-ecg-gated ct

## Abstract

Artificial intelligence (AI) is rapidly reshaping cardiology, with notable progress in calcium scoring for cardiovascular risk stratification. Emerging approaches now enable AI-driven scoring on non-ECG-gated chest CT, yet questions remain about comparative accuracy and real-world utility. This review synthesizes current work on AI applications in non-ECG-gated coronary artery calcium (CAC) scoring and subsequent cardiac risk assessment. We outline typical model inputs, development strategies, performance characteristics, and use cases across diverse care settings. Across the literature, AI-generated CAC scores generally show strong agreement with manual methods and support effective risk stratification, while markedly reducing processing time and workflow burden. Reported classifications align with clinically relevant outcomes, suggesting the potential to enhance the early identification of at-risk patients and streamline population-level screening. Despite promising results, heterogeneity in datasets, evaluation metrics, and deployment environments limits head-to-head comparisons and broad generalization. Implementation challenges, including image quality variation, integration with reporting pipelines, and oversight of edge cases, also warrant attention. Overall, AI for non-ECG-gated CAC scoring appears accurate, efficient, and clinically useful, with meaningful potential to improve cardiovascular risk assessment when thoughtfully validated and integrated into routine practice.

## Introduction and background

Background of the coronary artery calcium score

Atherosclerosis is a chronic inflammatory disease that involves the thickening and hardening of arteries due to plaque buildup on the inside of the vessels. It is the primary cause of atherosclerotic cardiovascular disease (ASCVD), which includes coronary heart disease (CHD), cerebrovascular disease, peripheral artery disease, and aortic atherosclerotic disease [1]. ASCVD is the most common cause of death worldwide, causing an estimated 19.8 million deaths globally in 2022, approximately 32% of global deaths [2]. Additionally, CVD is the leading cause of death in the US, with 680,981 deaths reported by the National Center for Health Statistics [3]. Common risk factors of atherosclerosis and ASCVD include diabetes, high blood pressure, family history of premature coronary artery disease, high cholesterol, older age, cigarette smoking, obesity, sedentary lifestyle, and diets high in saturated fats [3,4].

Plaque in atherosclerotic disease, composed of fat, cholesterol, calcium, and other substances, causes vessel lumen narrowing and can hinder blood flow [5]. The specific mechanism of plaque formation involves endothelial dysfunction, low-density lipoprotein (LDL) infiltration into the intima layer of vessels, and oxidation of LDL particles in the subendothelial compartment. These events trigger an inflammatory process involving macrophage recruitment and ingestion of oxidized LDL and cholesterol with subsequent transformation into foam cells and further atheroma plaque formation [3,6]. During plaque progression, microcalcifications are deposited through either the release of matrix vesicles from apoptotic macrophages or vascular smooth muscle cells (VSMCs), as well as VSMC differentiation into osteoblastic cells [6,7]. As the plaque grows, the calcifications can evolve and, thus, correlate with plaque size [6].

Given the high prevalence and clinical impact of ASCVD, it is critical to have proficient screening tools to identify patients at risk to implement life-saving preventative care. One of these tools is coronary calcium artery (CAC) scoring, which uses electrocardiogram (ECG)-gated computed tomography (CT) scans to measure the amount of calcium in the coronary arteries. Using ECG-gating allows imaging technology to capture images during periods of the cardiac cycle with less motion, thus limiting motion artifact that can hinder the accurate calculation of calcium deposition in the coronary arteries.

The Agatston method of CAC quantification

Traditional practice uses the Agatston method to quantify the degree of calcification by summing all the areas of increased attenuation in the coronary vessels. Specifically, this involves a manual-to-semi-automated process where radiologists confirm components in images greater than 130 Hounsfield units (HU) identified initially by software technology [8]. The calculated scores then allow patients to be stratified based on their risk of developing heart disease into the following groups: very low risk (CAC score of 0), mildly increased risk (CAC score of 1-99), moderately increased risk (CAC score of 100-299), and moderate to severely increased risk (CAC is greater or equal to 300) [9].

Rationale for non-ECG-gated analysis

Although ECG-gated CT scans offer the greatest precision in determining a patient's CAC score, there are existing limitations of these studies compared to the more commonly used non-ECG-gated chest CT scan. Research has shown that there are 0.5 million ECG-gated CT scans taken per year in the United States (US) compared to 10.6 million non-ECG-gated chest CT scans [10]. This is because non-gated chest CT scans are indicated in more clinical situations, including lung disease, infections, and lung cancer screening. In addition to the relatively less prevalent use of ECG-gated CT scans, insurance coverage for calcium scans is more difficult to obtain compared to their non-ECG-gated counterparts [11]. Thus, using non-ECG-gated CT chest scans can be the more readily available and cost-effective choice. In addition, using the common chest CT scan to calculate CAC scores provides an opportunity to collect multiple pieces of patient information with fewer imaging studies, thus reducing patients’ total exposure to radiation. If extensive atherosclerotic disease is detected in asymptomatic patients who are already undergoing a non-gated chest CT, these individuals can be protected from future cardiovascular events and medical costs [11].

The surge of artificial intelligence (AI) has been changing the landscape of the medical field and is showing promising applications in the field of CAC scoring with non-ECG-gated CT scans. Machine learning is a subset of AI that uses algorithms to analyze and learn from data sets with the help of human intervention to adjust the algorithm along the way. Deep learning is a subset of machine learning that uses artificial neural networks to automatically extract, process, and analyze information from large data sets, while independently learning from its own errors [12]. The deep learning technique that is mostly implemented in the automation of CAC scoring and medical image recognition is Convolutional Neural Networks (CNN) [13,14]. In basic terms, deep learning and machine learning programs are being taught to identify points of calcium deposition in chest CT images through the input of dense data sets consisting of thousands of non-gated chest CT scans.

Statement of purpose

This scoping review addressed the different methods used to develop novel deep learning models for CAC scoring from non-gated chest CT scans, as well as their accuracy and limitations compared to traditional methods. Using deep learning to calculate CAC scores from non-gated CT scans can change the future of cardiovascular disease screening. By leveraging data extraction from the more commonly used and readily available non-gated chest CT, more high-risk individuals can be identified to receive optimal and life-changing patient care.

## Review

Materials and methods

Eligibility Criteria

Articles that met the inclusion criteria needed to be accessible, peer-reviewed, primary studies in English that were published from January 2018 to September 2023. Additionally, they needed to focus on artificial intelligence-computed coronary artery calcification scores from non-ECG-gated scans. Articles that described calcification of other vessels and ECG-gated scans were not included.

Search Strategy

After agreeing on the inclusion criteria, the team consulted with a medical librarian to assist in determining a search strategy. Boolean operators were used to guarantee a comprehensive list of articles. The initial search strategy included the terms (“artificial intelligence” OR “machine learning” OR “deep learning” OR “sentiment analysis” OR “computational intelligence”) AND (“cac score” OR “calcium score” OR “calcium scans” OR “cardiac risk stratifications”). Table 1 depicts the search strategy using online databases.

**Table 1:** Database search translations. SCI: Science Citation Index; SSCI: Social Sciences Citation Index; CPCI-S: Conference Proceedings Citation Index-Science; CPCI-SSH: Conference Proceedings Citation Index-Social Science and Humanities; ESCI: Emerging Sources Citation Index; CCR: current chemical reactions; IC: Index Chemicus; TS: topic search; CAC score: coronary artery calcium score

No.	Database	Results
Query search strategy used in EMBASE (January 10, 2023)		
#1	'Artificial intelligence'/exp	86,206
#2	'Machine learning'/exp	418,866
#3	'Artificial intelligence':ab,ti,kw OR 'machine learning':ab,ti,kw OR 'deep learning':ab,ti,kw OR 'sentiment analysis':ab,ti,kw OR 'computational intelligence':ab,ti,kw	187,443
#4	'Coronary artery calcium score'/exp	8,742
#5	'Cac score':ab,ti,kw OR 'calcium score':ab,ti,kw OR 'calcium scane':ab,ti,kw OR 'cardiac risk stratifications':ab,ti,kw	12,185
#6	#1 OR #2 OR #3	475,796
#7	#4 OR #5	15,306
#8	#6 AND #7	537
#9	#8 AND (2018:py OR 2019:py OR 2020:py OR 2021:py OR 2022:py OR 2023:py)	487
Query search strategy used in Web of Science (SCI, SSCI, CPCI-S, CPCI-SSH, ESCI, CCR, IC) (January 10, 2023) results		
#1	TS=((“artificial intelligence” OR “machine learning” OR “deep learning” OR “sentiment analysis” OR “computational intelligence”))	612,673
#2	TS=((“cac score” OR “calcium score” OR “calcium scans” OR “cardiac risk stratifications”))	7,802
#3	#1 AND #2	214
#4	#1 AND #2 and 2023 or 2022 or 2021 or 2020 or 2019 or 2018 (publication years)	202
Query search strategy used in Ovid (MEDLINE) (January 10, 2023)		
#1	Artificial intelligence/	40,459
#2	("Artificial Intelligence" or "Machine learning" or "Deep Learning" or "Sentiment Analysis" or "Computational Intelligence").mp.	179,035
#3	("CAC Score" or "Calcium Score" or "Calcium Scans" or "Cardiac Risk Stratifications").mp.	6437
#4	1 or 2	179,035
#5	3 and 4	181
#6	Limit 5 to yr="2018 - 2023"	170

Study Selection and Screening Process

An initial search of three databases (Embase, Ovid MEDLINE, and Web of Science) identified 859 articles. Following the removal of 337 duplicates, 522 articles remained for screening. In tier 1 (title and abstract screening), studies were assessed against predefined eligibility criteria (full-text availability, peer-reviewed, primary research, English language, and publication between January 2018 and September 2023). This process excluded 270 articles, resulting in 252 articles remaining. Because the scope at this stage remained too broad, an additional inclusion criterion was applied as follows: studies must evaluate AI characterization of non-ECG-gated CT images. This refinement reduced the pool to 28 articles.

**Figure 1:**
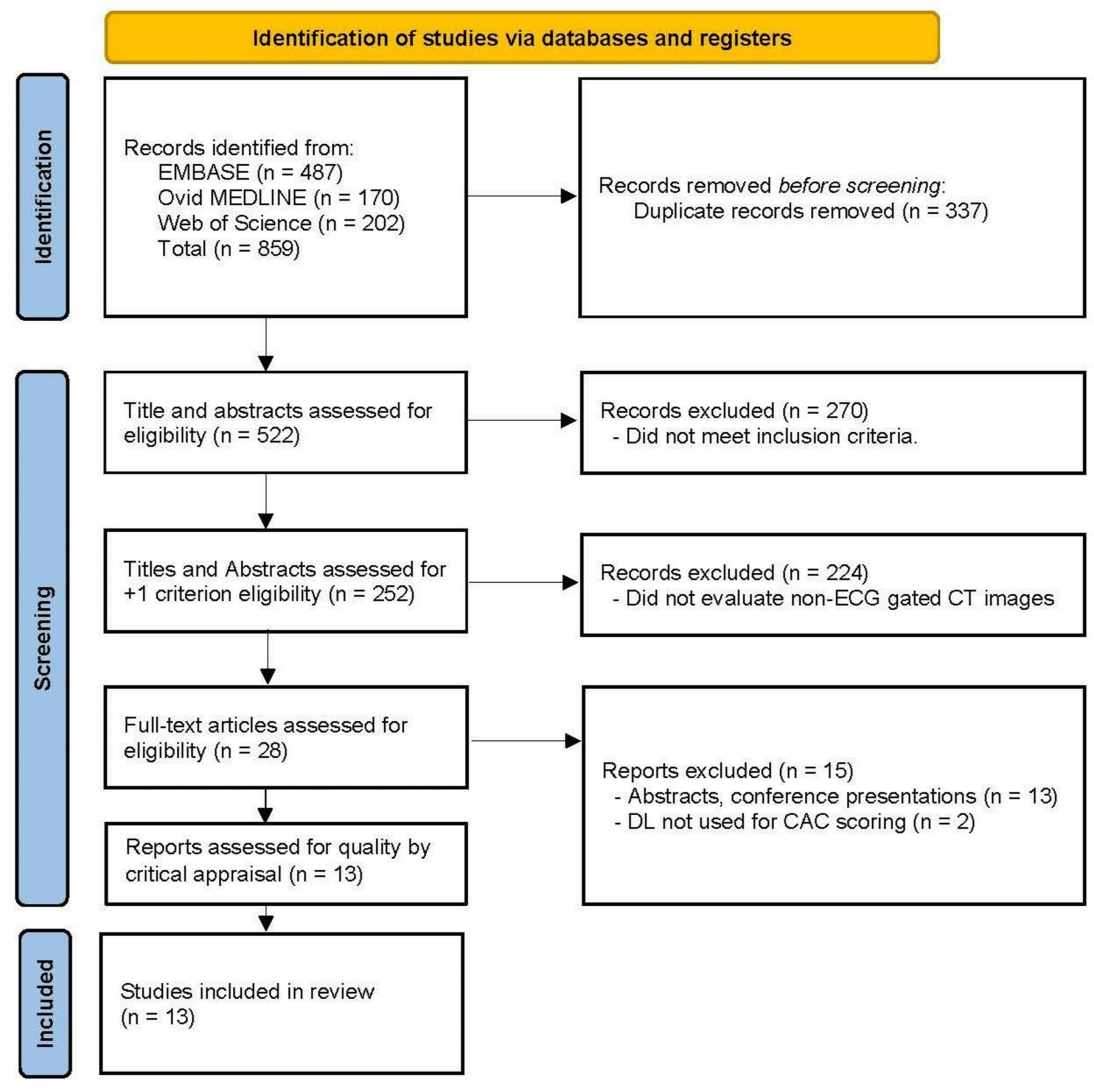
Preferred Reporting Items for Systematic Reviews and Meta-Analyses (PRISMA) flow diagram.

In tier 2 (full-text review), these 28 articles were further screened for eligibility. Of these, 13 were excluded because they were abstracts or conference presentations only, and two were excluded because the AI/DL algorithm was not applied to CAC scoring. This yielded 13 eligible studies (Figure 1).

Critical Appraisal of Individual Sources of Evidence

The 13 studies underwent quality and bias appraisal by two independent researchers using the Joanna Briggs Institute (JBI) Diagnostic Test Accuracy Studies critical appraisal checklists, which are free to access and download from JBI’s website (https://jbi.global/critical-appraisal-tools). As our review focused on articles analyzing the accuracy of a method for coronary artery calcium scoring, a tool designed to assess diagnostic test accuracy studies was selected for implementation within our scoping review research [14]. The utility of these tools lies in their ability to identify research bias, congruence, and imperative sections by which an article’s quality can be ascertained. Using this checklist, articles with a high risk of bias (scores <50%), moderate risk of bias (scores 50-70%), and low risk of bias (scores >70%) were identified by two team members. A final consensus was reached between the two researchers, and the final articles were selected (n=13).

Data Charting Process

Data were extracted from papers included in the scoping review by two independent reviewers using a data extraction tool developed by the reviewers. The data were charted independently by the two reviewers, and the results were discussed and updated as needed.

Data Items

Data were extracted on article characteristics (citation details), context (e.g., country and patient setting), participants (e.g., number, age, sex), study objectives, study design and setting, main findings, and limitations.

Synthesis of Results

Tables and diagrams were used to illustrate the data collected. These included the accuracy of models in scoring coronary calcium calcification and predicting risk of MI, types of AI models, numbers of model layers, and Agatston scores. A narrative summary accompanied each table, diagram, and chart to describe the results in the context of this review’s objectives and questions.

Results 

Study Selection and Participant Demographics

The initial search identified 859 records, of which 337 duplicates were removed. After title and abstract screening, 252 articles remained, which were narrowed to 28 based on the additional criterion of evaluating AI characterization of non-ECG-gated CT images. Following full-text review, 13 studies met all eligibility criteria and were included in the final review. All of these publications focused on testing commercially available deep learning technology or on developing deep learning technology to calculate CAC scores using data obtained from previous non-ECG-gated chest CT scans [15-27]. Out of the 13 articles, nine of these compared the automatic non-ECG-gated chest CT CAC scores calculated by the developed or existing deep learning technology to manually calculated scores using the gold standard Agatston equation [15-23], in addition to other comparison measures in a few cases [15,16,22,23]. The other four articles only compared the CAC scores generated from the AI technology interpretation of non-ECG-gated CT scans to CAC measurements calculated using measures different from the gold standard [24-27]. Of the articles that reported demographic information on their cohorts, the 13 studies consisted of a total of 25,446 patients (14,432 males and 10,784 females). Four cohorts had a mean age of 55-60 years, five cohorts had a mean age of 61-65 years, two cohorts had a mean age of 66-70 years, and one cohort had a mean age of 71-75 years.

AI Analysis of Non-gated Chest CT vs. Manual Analysis of Gated Cardiac CT (Gold Standard)

In the articles selected for summarization, nine of these compared automatic non-ECG-gated chest CT CAC scores statistics to the gold standard of manual ECG-gated cardiac CT CAC scores [15-23]. Absolute CAC score [15,17,19-21,23], CAC score risk stratification [15-23], and per vessel (left main {LM}, left anterior descending {LAD}, left circumflex {LCX}, right coronary artery {RCA}) CAC analysis were analyzed [15,17,19].

For the absolute CAC score, interclass correlation coefficients (ICC) and Spearman’s correlations were obtained in most of these. The absolute CAC score ICCs for the AI-non-ECG-gated CACs ranged from 0.714-0.955. The lowest ICC was obtained from an analysis of high CAC scores (>1000) [18]. Typically, “high-risk” CAC scores are considered >300-400, and most of the articles examine CAC scores in that 0-400 range [15-17,19,21,22]. In this range, the lowest ICC is 0.88, which is generally a much better coefficient than 0.714 [19]. Spearman’s correlations demonstrated a very strong correlation and ranged from 0.893 to 0.95 [17,21]. Specificity was also examined in one study, where a range of 83-86% specificity was demonstrated [20].

For CAC score risk stratification, weighted Kappa scores comparing AI categorization of CAC as low, moderate, or high via analysis of non-ECG-gated chest CT vs. manual categorization of CAC via analysis of ECG-gated cardiac CT were used for analysis of AI’s consistency with the gold standard in most studies [15-17,19-22]. Weighted Kappa scores ranged from 0.5 to 0.84 [16,22]. These demonstrate moderate to significant correlation between the AI and manual methods of analysis of the differing CT scans. It is worth noting that across the different studies, the misclassified scans by AI fell largely within one category of correct and mostly underestimated the correct category [16,17,19,21]. Although one article did demonstrate a tendency of AI overestimating the CAC and misclassifying it to a higher category more often than a lower category [20]. The specificity of risk classification was a strong point for the AI models. While their specificity ranged from 59.9 to 100%, the AI model’s specificity tended to remain in the 90th-100th percentile range [15,16,22]. One study demonstrated an area under the curve (AUC) of 0.881 when AI was used to classify a non-gated chest CT scan as either greater or less than 400 CAC for high-risk screening compared to manual analysis of ECG-gated cardiac CT [23].

ICC coefficients, Spearman’s correlations, weighted Kappa scores, and diagnostic statistics (sensitivity/specificity) were used for the per-vessel analysis of the AI interpretation of non-ECG-gated chest CT scans vs. manual interpretation of ECG-gated cardiac CT [15,17,19]. Per vessel ICCs ranged from 0.716 to 0.867 for the left main (LM), 0.863 to 0.928 for the left anterior descending (LAD), 0.716 to 0.927 for the left circumflex (LCX), and 0.812 to 0.944 for the right coronary artery (RCA) [15,19]. Spearman’s correlations were calculated at p=0.58 for the LM, p=0.88 for the LAD, p=0.80 for the LCX, and p=0.81 for the RCA [17]. Sensitivities ranged from 40.7 to 74.5% for the LM, 82.0 to 82.2% for the LAD, 64.2 to 65.1% for the LCX, and 61.7 to 65.3% for the RCA [17,19]. Specificities remained high and ranged from 79.6 to 99.2% for the LM, 98.4 to 100% for the LAD, 95.7 to 99.4% for the LCX, and 95.0 to 100% for the RCA [17,19].

AI Analysis of Non-gated/gated CT vs. Other Measures

Reliability of the AI model was established via analysis of CAC scores produced by AI analysis of non-ECG-gated chest CT scans compared to manual analysis of non-ECG-gated chest CT scans, in addition to analysis of CAC scores produced by AI analysis of ECG-gated cardiac CT scans compared to manual analysis of ECG-gated cardiac CT scans. Non-ECG-gated reliability was established with ICC coefficients, weighted Kappa scores, and Spearman’s correlations. Overall ICC coefficients ranged from 0.989 to 0.992 with per vessel ranges of 0.522-0.863 for the LM, 0.964-0.975 for the LAD, 0.962-0.993 for the LCX, and 0.980-0.983 for the RCA [24,25]. Weighted Kappa scores ranged from 0.855 to 0.946 overall [24,25]. Spearman’s correlations were calculated to 0.977 total, 0.736 for the LM, 0.906 for the LAD, 0.897 for the LCX, and 0.935 for the RCA [25]. ECG-gated reliability was established with ICC coefficients, weighted Kappa scores, and diagnostic statistics (sensitivity/specificity). ICCs ranged from 0.998 to 1.000 total and per-vessels ranging from 0.928 to 0.944 for the LM, 0.994 to 0.995 for the LAD, 0.992 to 0.995 for the LCX, and 0.995 to 1.000 for the RCA [15,24]. Retrospective weighted Kappa scores ranged from 0.89 to 0.975 [15,16,22,24]. Prospective weighted Kappa scoring was calculated at 0.83 [22]. Cutoff score sensitivities/specificities were calculated to 100%/93% for CAC 1, 98.9%/99.0% for CAC 10, 99.4%/100% for CAC 100, and 100%/100% for CAC 400 [15].

AI scoring of both non-ECG-gated chest CTs and ECG-gated cardiac CTs was demonstrated to be linked to morbidity and mortality. AI CAC scoring of non-ECG-gated chest CTs was demonstrated to be capable of screening for CVD with an AUC=0.924 (95% CI: 0.909-0.940) and additionally could high-low risk stratify compared to manual ECG-gated cardiac CT calculated CAD-RADS scores, AUC=0.763 [23]. AI CAC scoring of non-ECG-gated CTs was also demonstrated to be able to predict CVD mortality risk, AUC=0.768 [23]. These scores were also linked to increasing hazard ratios of CVD compared to the reference very low category when calculating CAC categories (1-100, 101-300, >300) with hazard ratios of 1.57, 2.79, and 3.87 for each category, respectively [26]. When AI performed CAC scoring on non-ECG-gated chest CT, higher CAC scores were associated with significantly higher (p<0.001) MACE rates, irrespective of ischemia (p<0.001), were a significant univariable predictor of major adverse cardiac events (MACE) (HR: 3.49, 95% CI: 2.41-4.60), and were independently associated with MACE (HR: 2.26 95% CI: 1.48-3.46) compared to lower CAC scores [27].

Finally, the average time for CAC processing via AI was demonstrably shorter than that of the gold standard manual method, at 3.5±2.1 s compared to 261 s on average [22]. A summary of the articles in this review is displayed in Table 2.

**Table 2:** Summary of selected literature for this review (n=13). CT: computed tomography; LDCT: low-dose computed tomography; CSCT: calcium scoring computed tomography; PET: positron emission tomography; PET/CT: positron emission tomography/computed tomography; MPI: myocardial perfusion imaging; SPECT: single-photon emission computed tomography; SPECT-MPI: single-photon emission computed tomography-myocardial perfusion imaging; 82Rb-PET/CT: rubidium-82 positron emission tomography/computed tomography; 18F-FDG PET/CT: fluorine-18 fluorodeoxyglucose positron emission tomography/computed tomography; N-13 ammonia PET: nitrogen-13 ammonia positron emission tomography; CAC: coronary artery calcium; CACS: coronary artery calcium score; CAD: coronary artery disease; CVD: cardiovascular disease; MACE: major adverse cardiac events; CV: cardiovascular; h/o: history of; LM: left main (coronary artery); LAD: left anterior descending (coronary artery); LCX: left circumflex (coronary artery); RCA: right coronary artery; AI: artificial intelligence; DL: deep learning; AI-CACS: artificial intelligence-based coronary artery calcium scoring; AVIEW: commercial software tool for automated CAC scoring; FN: false negative; TP: true positive; FP: false positive; ICC: intra-class correlation coefficient; NPV: negative predictive value; PPV: positive predictive value; AUC: area under the curve; MESA: multi-ethnic study of atherosclerosis; NLST: National Lung Screening Trial; FHS-CT: Framingham heart study-computed tomography; PROMISE: prospective multicenter imaging study for evaluation of chest pain; ROMICAT-II: rule out myocardial infarction using computer-assisted tomography II; MYOMARKER trial: prospective imaging trial using Rb-82 PET/CT; ECG: electrocardiogram (used in ECG-gated CT); HIS: hospital information system; ICD-10: International Classification of Diseases, 10th Revision AVIEW software (Seoul, South Korea: Coreline Soft Co., Ltd.)

Studies	Title	Year	Country of origin	Participants	Study setting	Study design	Study objectives	Main findings	Limitations
Kang et al. [15]	Evaluation of fully automated commercial software for Agatston calcium scoring on non-ECG-gated low-dose chest CT with different slice thickness.	2023	Germany	567 participants, 343 males and 224 females, mean age of 58.3±11.1 years.	Retrospective study with participants who underwent non-electrocardiogram (ECG)-gated low-dose CT (LDCT) and ECG-gated calcium-scoring CT (CSCT) from July 2019 through January 2022 at one institution.	Retrospective, participants underwent paired CSCT and LDCT within six months at the same institution. LDCT images were reconstructed with 2.5 mm and 1.0 mm slice thicknesses. Manual analysis of the CSCT was performed by one experienced technologist with review from an experienced CT radiologist. CSCT and LDCT (2.5, 1.0) volumes were analyzed separately by a commercial DL AVIEW software algorithm, and analysis of whole-heart and differential vessel analysis was performed.	To assess commercial deep learning-based software (DL AVIEW) for fully automatic Agatston scoring in LDCT with differential slice-thickness compared to gold standard CAC scoring in CSCT.	Total CAC scores and individual vessel scores when CSCT auto, LDCT 1 mm auto, and LDCT 2.5 mm auto all demonstrated excellent agreement with CSCT manual. Interclass correlation coefficients (ICCs) of LDCT 1 mm auto for LAD, LCX, and RCA were all higher than LDCT 2.5 mm auto. ICCs for LDCT 1 mm auto showed higher ICCs with CSCT across all groups when compared to LDCT 2.5 mm auto. The Kappa agreements between the CSCT auto and LDCT 1 mm auto, compared to CSCT manual, were almost perfect (0.975, 0.809), while LDCT 2.5 mm auto was substantial when compared to CSCT manual (0.776). Additionally, CSCT auto, LDCT 2.5 mm auto, and LDCT 1 mm auto only had equal to or more than 2 category difference rates of 0.3%, 5.6%, and 0.7% respectively. Overall, there was an underestimation of CACs in all the LDCT analyses compared to CSCTs, but they were smaller in the LDCT 1 mm analyses compared to the LDCT 2.5 mm.	Retrospective, post-CT processing required for analysis, smaller data set.
Dobrolinska et al. [16]	Performance of visual, manual, and automatic coronary calcium scoring of cardiac N-13-ammonia PET/low-dose CT.	2023	Netherlands	213 participants, demographic information was obtained for only 174 of these: 95 male and 79 female, mean age of 61.7±9.3 years.	Retrospective study with participants who underwent both an LDCT with 13N-ammonia PET myocardial perfusion imaging (MPI) and a dedicated CSCT scan between 2013 and 2019.	Retrospective analysis of both LDCTs and CSCTs from symptomatic or h/o of coronary artery disease (CAD) participants who received a 13N-ammonia PET MPI with LDCT. All images (both LDCT and CSCT) were visually, manually, and automatically CAC-scored. Manual scorings were performed according to the Agatston method by two experienced observers. Automatic scorings were performed by a commercially available algorithm. Visual scoring was performed twice by one observer blinded to the results of the gold standard.	To compare automatic, manual, and visual CAC scoring performance between LDCT images acquired from 13N-ammonia PET MPI LDCT images against manual scoring of dedicated gold standard CSCT images.	Automatic and manual CSCT CAC scores were similar, with excellent correlation and excellent ICCs. Both automatic and manual LDCT CAC scores significantly underestimated the CAC score compared to the gold standard (manual CSCT). While the correlation for automatic scoring was excellent, 12.7% of automatic LDCT CACs were incorrectly assigned to the zero-category, demonstrating poor negative predictive value (NPV) at 30.8%. However, the positive predictive value (PPV) of automatic LDCT CACs was 100%. 4.2% of manual LDCT CACs were incorrectly assigned to the zero-category, demonstrating an NPV of 51.7% for manual LDCT CAC compared to the gold standard. Visual LDCT assessment performed better than either automatic or manual CSCT CAC assessment, and interobserver agreement in visual assessment was high (Kappa 0.94).	Retrospective, the deep learning model was not trained on LDCT images but rather was commercially available for CSCT scan analysis, and does not compare automatic LDCT analysis to manual LDCT analysis.
Yu et al. [17]	Automated total and vessel-specific coronary artery calcium (CAC) quantification on chest CT: direct comparison with CAC scoring on non-contrast cardiac CT.	2022	China	405 participants, 235 males and 170 females, mean age of 59.6±11.8 years.	Retrospective study including data from gated and non-gated chest CTs from August 1, 2019, to December 31, 2020.	Retrospective analysis of participants who underwent both gated cardiac CT and non-gated chest CT for lung cancer screening within 0-18 days of each other. Gated cardiac CTs were manually CAC-scored by experienced technicians. Non-gated chest CTs were CAC-scored via both automatic and manual assessment. Additionally, vessels were identified and analyzed individually.	To assess the performance of automated quantification of the CAC score and risk categorization, in addition to vessel-specific CAC assessment on non-gated CT images, compared to CAC scoring on ECG-gated cardiac CT as a standard.	Automated CACs showed moderate reliability for the number of involved vessels in comparison to that on dedicated cardiac CT (Kappa of 0.75). Per vessel automated analysis demonstrated poor sensitivity compared to specificity. Motion artifact was the primary cause of false negatives. In participants with positive CAC, the correlation between non-gated and gated CAC was strong. CAC was significantly underestimated for all participants in each risk group (47 underestimates in comparison to seven overestimates).	Retrospective, small sample size, thin imaging slices.
Sartoretti et al. [18]	Opportunistic deep learning powered calcium scoring in oncologic patients with very high coronary artery calcium (≥1000) undergoing 18F-FDG PET/CT.	2022	United Kingdom	100 participants, 50 in the control (<1000 CAC) group (30 males, 20 females; mean age of 71±7 years), 50 in the test (≥1000 CAC) group (40 males, 10 females; mean age of 70±8.9 years).	Retrospective study of consecutive participants undergoing whole-body 18F-FDG-PET/CT and MPI with various malignant diseases at the University Hospital Zurich, Switzerland, between November 2007 and February 2015.	Retrospective cohort of consecutive participants who underwent both 18F-FDG-PET/CT scans and underwent one-day stress/rest MPI with 99-mTc-tetrofosmin SPECT-MPI, including a non-contrast ECG-gated CT scan for attenuation. Scans were performed within six months of each other. Participants with ≥1000 CAC were included, and manual CAC scoring was performed on the ECG-gated CT scans. Participants with <1000 CAC were included as "negative" controls, with manual CAC scoring being performed on these by ECG-gated CT analysis as well. A cloud-based DL tool analysis was performed on non-ECG-gated CTs from all participants. Researchers were looking for congruence of analysis between the manual and automatic scoring of "positive" (≥1000 CAC) and "negative" (<1000 CAC) scans. No training data were included in the study.	To assess the feasibility of identifying very high CAC by means of DL powered CAC scoring in oncologic participants with known very high coronary artery calcium scores (≥1000) undergoing 18F-FDG-PET/CT.	The automatic method generally performed the analysis of CACS faster than the human counterpart. The average CAC score for the test cohort manually scored scans was 2200±1620 compared to 1300±1011 by the DL tool analysis. The CAC score was generally underestimated by the DL tool in 90% of test cases. When outcomes were binarized as <1000 or ≥1000 in the test cohort, the DL tool assigned <1000 in 23/50 cases (false negative {FN} rate of 46%) and ≥1000 in 27/50 cases (true positive {TP} rate of 54%). When the test cohort was assessed and separated for image quality as insufficient/sufficient (scores of 1-2 and 3-4, respectively), the DL tool scored "sufficient" image quality scans at 71.4% TP and 28.6% FN rates compared to "insufficient" quality scans at 31.8% TP and 68.2% FN rates.	Small cohorts, retrospective, cloud-based tool, lack of DL tool training, and potential six-month difference in non-gated and gated CT scans.
Morf et al. [19]	Diagnostic value of fully automated artificial intelligence-powered coronary artery calcium scoring from 18F-FDG PET/CT.	2022	Switzerland	100 participants, 66 males and 34 females, 66±11.4 years.	Retrospective cohort study of consecutive participants undergoing both 18F-FDG-PET/CT and 99mTc-tetrofosmin SPECT-CT MPI with non-contrast, ECG-gated CT for attenuation correction within six months of 18F-FDG-PET/CT imaging with malignant disorders and known/suspected CAD at the University Hospital of Zurich between November 2007 and February 2015.	Retrospective cohort study of consecutive participants undergoing (a) a whole-body 18F-FDG-PET/CT, and (b) 1-day stress/rest myocardial perfusion imaging by 99mTc-tetrofosmin SPECT-MPI with ECG-gated CT for attenuation correction within 6 months of 18F-FDG-PET/CT. Automated (AI-CACS) CAC scores were obtained from 18F-FDG-PET/CT imaging and compared to manual CAC scores obtained from dedicated ECG-gated CT scans as obtained during MPI. Two experienced physicians in consensus measured the manual scores. No training data were included in this study, thus representing an external validation of the automatic AI-CACS algorithm.	To assess the quantitative accuracy of an AI-powered tool (AI-CACS) in estimating CAC from CT scans acquired during 18F-FDG PET/CT examinations using manual CACS measurements from a dedicated cardiac imaging workup as a standard of reference.	AI-CACS demonstrated a sensitivity/specificity of 85%/90.0% total and per vessel of 74.5%/79.6% LM, 82%/100% LAD, 64.2%/95.7% LCX, 61.7%95.0% RCA when compared to manual reference. AI-CACS was more prone to false negatives than false positives. Inter-score agreement between AI and manual reference was 0.88 overall, and interclass agreement was 0.80. AI-CACS analysis of ungated CT generally underestimated the CAC burden.	Small data set, retrospective, tool had been previously validated on dedicated cardiac calcium CT scans with good agreement/performance, but not on non-gated CT scans. The tool was used as is without further optimization by the research team, six-month time difference between non-gated and gated CT scans.
Xu et al. [20]	Automatic coronary artery calcium scoring on routine chest computed tomography (CT): comparison of a deep learning algorithm and a dedicated calcium scoring CT.	2022	Hong Kong	Internal cohort: 406 participants, 217 males and 189 females, mean age 61.8±12.1 years. External cohort: 117 participants, 74 males and 43 females; mean age 59.8±12.6 years.	Retrospective study performed at two tertiary hospitals.	Retrospective study of participants with paired non-ECG-gated and ECG-gated CT scans. Gated CT scans were manually CAC-scored by radiologists. The thickness of CT slices was varied from 1 mm to 5 mm slice thickness at 1 mm and 3 mm increments. Non-gated CT scans were scored automatically and manually assessed, and a 22-layer DL algorithm was produced.	To propose and validate a two-stage segmentation DL algorithm and to investigate the reliability and accuracy of the automatic CAC scoring and risk classification on non-gated chest CT scans with differential slice thicknesses.	Reliabilities of the algorithm were good, with 3 mm mean differences on LDCT and CSCT being slightly better than 1 mm. There were systematic differences in both 1 mm and 3 mm slice increments. There were excellent ICCs between the automatic and gold standard. Agreement between the automatic and gold standards was good, with a Kappa value of 0.72. External validation demonstrated a 0.80 Kappa value for 1 mm and a 0.80 Kappa value for 3 mm for CAC categorization.	Retrospective, small sample size, DL algorithm tended to overestimate risk category, susceptible to motion or metal artifact.
Xu et al. [21]	Performance of artificial intelligence-based coronary artery calcium scoring in non-gated chest CT.	2021	China	901 participants, 518 males and 383 females, mean age of 62.9±22.9 years.	Retrospective study with baseline characteristics collected from the Hospital Information System (HIS) of Hospital of Chongqing Medical University between November 2018 and January 2021.	Retrospective study of participants who underwent both non-gated and gated chest CT scans within three months. Automated CAC scores were obtained from non-gated chest CT images, and manual CAC scores were obtained from the gated chest CT images.	To assess the correlation between automated CAC scoring of chest CT and manual CAC scoring of cardiac CT, and to confirm risk category performance considering manual CACS as standard.	Automated CAC score agreement with the manual CAC score was assessed with a Kappa coefficient of 0.679. There were 158 cases of automated CAC score underestimation and 17 cases of overestimation. Most underestimated cases belonged to the very low to low-risk groups. The risk categorization analysis of the automated CAC score with the manual CAC score was good, with a 0.77 Kappa coefficient.	Retrospective, compared only the DL analysis of LDCT to the manual analysis of CSCT and no hybrids, motion artifact, three-month time interval between LDCT and CSCT scans.
Eng et al. [22]	Automated coronary calcium scoring using deep learning with multicenter external validation.	2021	United Kingdom	Non-gated model Cohorts: (A) Stanford: 42 participants, 24 males and 18 females, mean age of 60.6±7.5 years. (B) MESA: 46 participants, 21 males, 24 females, and 1 "unavailable", mean age of 65.9±9.0 years. (C) External validations: Site 1 (22 participants, 22 males and 0 females, mean age of 64.6±10.1 years), Site 2 (75 participants, 53 males and 22 females, mean age of 62.4±13.3 years), Site 3 (71 participants, 46 males and 25 females, mean age of 66.5±9.0 years), Site 4 (135 participants, 61 males and 74 females, unavailable mean age).	Retrospective study with retrospective validation of developed automated models. Participants were divided into three cohorts by source of CT scan images. Group A had non-gated CT scans obtained from Stanford Hospital, Group B had scan information from the Multi-Ethnic Study of Atherosclerosis (MESA) study, and group C had external site validations from four different sites.	The developed automatic models were first tested on retrospective and prospective gated test sets and compared to manual scoring. These were then internally validated on two retrospective non-gated data sets that had paired gated data. These were then externally validated on retrospective non-gated data sets from three sites with paired gated data.	To propose two developed DL models that automatically quantify vessel-specific CAC score using ECG-gated and non-ECG-gated CT.	Developed automatic models demonstrated little difference in gated CAC analysis compared to manual scoring in retrospective and prospective analysis. Developed models demonstrated almost perfect agreement in bucketed CSCT scores when compared to manual CSCT (Kappa of 0.84) on internal non-gated validation. Developed models demonstrated substantial agreement in bucketed scores compared to manual CSCT (Kappa coefficients of 0.68, 0.64, 0.58) on external non-gated validation.	New DL model development, limited/small datasets.
Chao et al. [23]	Deep learning predicts cardiovascular disease risks from lung cancer screening using low-dose computed tomography.	2021	United States	335 participants, 161 males, 174 females, mean age of 63.6±8.0 years.	Retrospective study with data from both the NLST study trial and the Massachusetts General Hospital (MGH) on participants who underwent low-dose computed tomography (LGDT) in 2020.	Retrospective training, evaluation, and validation of a proposed deep neural network model using the NLST study dataset with mortality data randomly split into three cohorts (training, validation, testing). An additional independent dataset was used for validation. Participants were pre-identified as CVD positive or negative based on medical history, cardiovascular abnormalities on CT, cardiovascular disease reporting, and mortality outcomes. CVD “positive” and “negative” participants from the NLST study were randomized and split into the three cohorts. Three radiologists performed CAC score grading on the NLST images.	To propose an end-to-end deep neural network to (a) screen participants for CVD and (b) quantify CVD mortality risk (CAC scores) directly from LDCT scans.	The proposed model identified CVD positive vs. negative participants with significant area under the curve (AUC) accuracy (0.87 screening, 0.76 mortality). This AUC accuracy was better than other proposed methods for LDCT and similar to radiologist CAC grading in the NLST dataset. The model also demonstrated significant AUC accuracy (0.924 screening, 0.881 CAC score, 0.763 CAC-RADS, 0.835 MESA) in stratifying participants as high or low CVD risk based on the LDCT scans only, compared to other proposed methods for LDCT in the MGH dataset, in addition to the comparison to cardiologist calculations from dedicated CT scans.	Retrospective, only evaluated cardiovascular outcomes and CVD presence rather than CAC quantification, used only ICD-10 CVD diagnoses and causes of death to validate the model against which could have missed CVD cases.
Suh et al. [24]	Fully automatic coronary calcium scoring in non-ECG-gated low-dose chest CT: comparison with ECG-gated cardiac CT.	2023	Germany	452 participants, 384 males, 68 females, average age of 55.6±9.8 years.	A retrospective study that included subjects from three academic institutions.	Three retrospective cohorts from different geographic locations, who had both same-day or within six months non-gated LDCT and gated CSCT performed, were enrolled. Automatic CAC score was acquired on both gated and non-gated CT scans using AVIEW commercial software. Manual CAC score was acquired from both gated and non-gated CT scans by two expert radiologists. CAC score total and per-lesion analyses were performed on all CT scans.	To validate an AVIEW AI-based fully automatic CAC scoring system on non-ECG-gated LDCT using multi-institutional datasets with manually segmented CAC scoring as the reference standard.	Automatic CAC of gated CT images was compared to manual CAC on the same images to determine reliability. ICCs of 0.998 and per-vessel evaluation ICCs of 0.928 LM, 0.994 LAD, 0.992 LCX, 0.995 RCA, thus demonstrating high reliability of the AVIEW algorithm. CAC auto demonstrated excellent reliability with CAC auto of LDCT, assigning 85% of subjects to the correct risk category and 12.6% to a neighboring category. In per-lesion analysis, CAC auto yielded 0.58 per-subject per-lesion FP rate on CSCT compared to manual, and a 0.38 per-subject per-lesion FP rate on LDCT compared to manual.	Retrospective, small sample size, commercial algorithm not trained on LDCT, noise/artifact.
Choi et al. [25]	Validation of deep learning-based fully automated coronary artery calcium scoring using non-ECG-gated chest CT in patients with cancer.	2022	South Korea	913 participants, 633 males and 280 females; mean age of 68.3±10.6 years.	Retrospective study with participants diagnosed with colorectal or gastric cancer with existing baseline chest CT scans, including non-contrast-enhanced CT images, between 2013 and 2015 at Chung-Ang University Hospital.	Retrospective analysis of non-ECG-gated chest CT scans using AVIEW CAC DL automatic CAC detection, quantification, and categorization software with intra-class coefficients of both the general heart and individual vessels. This was referenced or proved against an experienced technologist's Agatston method interpretation of the same total heart and individual vessels using the same unenhanced non-gated chest CT images.	To validate a recently released deep learning algorithm-based CAC scoring software for non-ECG-gated chest CT scans from cancer participants to demonstrate reliability and clinical applicability.	The DL algorithm demonstrates high intra-class correlation (ICCs of 0.992 total, 0.863 LM, 0.964 LAD, 0.962 LCX, 0.980 RCA) to experienced technologist CAC interpretations of the same non-ECG-gated chest CT scans. 42 of 44 misclassifications by the DL algorithm were placed in an adjacent category.	Retrospective, lacked ECG-gated CAC scoring CTs as reference, only the Agatston method was evaluated and no other methods of CAC classification, cardiovascular outcomes not investigated.
Zeleznik et al. [26]	Deep convolutional neural networks to predict cardiovascular risk from computed tomography.	2021	United States	20,084 participants, 11,320 males and 8,764 females, mean age of 61.0±6.0 years.	Retrospective secondary analysis of a longitudinal primary prevention cohort (FHS-CT1 and FHS-CT2) and three randomized clinical trials (NLST, PROMISE, ROMICAT-II).	Retrospective analysis of FHS, NLST, PROMIS, and ROMICAT trial data. The DL system was developed and tested on gated cardiac and non-gated chest CT scans from these trials. The association between DL CAC scores derived from NLST LDCT images and the incidence of atherosclerotic CVD was investigated. The DL algorithm was tested against the symptomatic PROMISE ECG-gated trial data. All DL CAC scores from gated and non-gated CT scans were compared to manual CACs from these studies.	To propose an automated model that automatically and accurately predicts CV events by quantifying the presence of CAC.	Automated AI/DL-based models were able to accurately stratify risk for cardiovascular events across 20,084 individuals in both non-gated and gated cardiac CT. It also demonstrated high correlation with human expert readers and robust test-retest reliability. The DL CAC score was shown to be an independent predictor of adverse cardiovascular events in all cohorts.	Retrospective analysis of trial data instead of new data.
Dekker et al. [27]	The prognostic value of automated coronary calcium derived by a deep learning approach on non-ECG-gated CT images from 82Rb-PET/CT myocardial perfusion imaging.	2021	Netherlands	747 participants, 377 males and 370 females, mean age of 67±10 years.	Prospective single-center observational cohort study of consecutively enrolled outpatient clinic participants aged >18 years with suspected symptomatic CAD between August 2014 and September 2016 at Meander Medical Center (General Hospital).	Retrospective analysis of the MyoMarker prospective 82Rb-PET/CT MPI imaging trial. LDCT scans were CAC-scored via an automated DL algorithm and stratified as "ischemic" or "non-ischemic" classifications of PET/CT imaging from the trial. These were then correlated to cardiovascular outcome, and the CAC score trend was compared to the major adverse cardiac events (MACE) trend.	To assess the correlation of automatic (DL acquired) CAC acquisition from myocardial perfusion imaging and LDCT (non-gated) scans with the occurrence of MACE.	Both ischemia and high CAC scores obtained from automatic LDCT analysis were demonstrated as univariate predictors of MACE.	Retrospective, does not evaluate the accuracy of the automatic categorization of CAC scores.

Discussion

Rationale for Clinical Utility of AI Interpretations of Non-ECG-Gated Chest CT Images

The CAC score is used as a prognostic factor in cardiovascular disease. There are three important CAC cutoffs over which management guidelines suggest escalating intervention. The first of these is a score of 0, under which is the most significantly correlated negative-risk predictor in CVD [28,29]. The next of these is a score of 100, over which it is a strong recommendation to initiate statin therapy to reduce the risk of CVD-related complications [30]. The final of these is a score of 400, over which it has been recommended that invasive coronary angiography be performed instead of coronary CT angiography, as it is believed to be uninformative [31]. Due to these significant prognostic markers, it has been recommended by the Society of Cardiovascular Computed Tomography, Society of Thoracic Radiology, and British Societies of Cardiovascular Imaging/Cardiac Computed Tomography and Thoracic Imaging that incidental CACs and coronary calcifications be reported on all chest CT scans [32,33]. Although the importance of calculating and reporting the CACs on as many patient chest CTs as possible has been underlined and emphasized, practice has yet to catch up to the recommendations. In 2014, 19 million non-gated chest CT scans were performed for non-cardiac indications [34,35]. However, almost 80% of these radiologists' reports made no mention of coronary calcifications [36,37]. While upwards of 80% could have truly been negative for coronary arterial calcification, the prevalence of cardiovascular disease and atherosclerosis could indicate that this is an under-reported figure. Several factors contribute to the overall cost of coronary arterial calcification characterization, including space, software, and technologist salaries, among others [38,39]. These collectively suggest that the cost associated with manual characterization is most likely comparable, if not in excess, of the costs associated with maintaining an integrated automated (AI) CAC tool for non-ECG chest CT analysis. Additionally, it has been shown that the average time required for CAC processing via the automated method is significantly faster than that of the gold standard method [22]. Consequently, serious research into the feasibility of producing a well-performing AI tool for these purposes may warrant serious consideration by the medical community.

Review of Results and Interpretation

The primary question of concern that this scoping review aimed to answer was how well these automated methods of CAC scoring, using non-ECG-gated chest CT, perform in comparison to the gold standard methods. Sub-questions that warranted answering in addition were what the reliability of automated non-ECG-gated chest CT CAC scoring is relative to human scoring of the same CTs, and whether CAC scores computed from automated analysis of these same CTs are linked to morbidity and mortality. Overall, according to all of the articles included in this scoping review that compare automated CAC methods to the gold standard, automated CAC scoring methods implemented on non-ECG-gated chest CT scans, by and large, underestimate the CAC score [15-23]. This underestimation tends to increase as CAC scores increase, with the largest underestimations seen in the 1000+ scoring categories [18]. This underestimation when comparing risk stratification levels (0, 10, 100, 300+) tends to almost always (upwards of 89%) fall short by only one risk category [19]. That being said, due largely to motion artifact, the sensitivity of these automated methods on these non-gated CT scans tends to be particularly poor, with many more false negatives even compared to automated methods used on dedicated ECG-gated cardiac CT scans [16]. While this performance may not seem the greatest, it is largely comparable to human performance on the same non-ECG-gated chest CTs, with the biggest difference being that humans had significantly fewer false negatives (4.2%) on the same CTs compared to the automated methods (12.7%) [16]. This is relatively poor performance considering that a score of 0, as stated earlier, is widely considered to be the largest negative-risk predictive value in the prognosis of cardiovascular disease [28,29]. On the other hand, this high reliability of the automated CAC scoring on non-ECG-gated chest CTs compared to manual CAC scoring of the same CTs (ICC >0.9, weighted Kappa=0.748-0.924) should not be understated [24]. Finally, the fact that increasing automated CAC scores obtained from non-ECG-gated chest CTs are a significant univariate predictor of MACE (HR: 3.49, 95%CI: 2.41-4.60) compared to lower CAC scores should support the idea of treating patients for CVD risk dependent on their high automated-CAC scores, irrespective of potential underestimation [27].

Rationale for a Potential Niche of AI in CAC Scoring and Radiology in General

While the limited literature available may support the reporting of automated CAC scoring on all chest CTs regardless of ECG-gating status due to the automated methods' high reliability compared to human interpretation, the particularly dismal sensitivities and negative predictive values of the automated method of scoring, 81.7% and 30.8% respectively, negates the use of this method as a means of screening for 0 CAC score risk as currently developed [16]. On the other hand, manual interpretation and reporting of the same non-ECG-gated chest CTs is encouraged, even considering the similar underestimation of manual and automated scoring of the same non-ECG-gated chest CTs [32,33]. A potential niche that reporting of these automated scores could inhabit is that of acting as an indicator of medical necessity for performing a dedicated ECG-gated cardiac CT. This could be useful because currently, it is largely hard to acquire reimbursement from insurance for gold standard CAC scoring without active, symptomatic cardiovascular disease [22]. Replacing the gold standard of CAC scoring is not the place of this automated method of CAC from non-ECG-gated CT scans. However, identification and preventative management of at-risk asymptomatic individuals for CVD without increasing their radiation dose by having to be scanned again for the dedicated CAC cardiac CT scan is an invaluable factor and constitutes true, patient-centered care.

This review set out to compare the use of AI in calculating non-ECG-gated CAC scores with manually calculated scores. The results revealed that overall, outcomes derived from both methodologies were comparable. AI can indeed be a useful tool for cardiac risk assessment due to its reliability, as displayed by the articles reviewed. Furthermore, with the potential for increased efficiency in AI-generated risk stratification, more patients can benefit from such technology. Furthermore, the use of AI does not exclude the importance of qualified radiologists overseeing the process. Rather, it may help providers make more accurate diagnoses with the help of a second source of judgment.

Statement of Relevance

This scoping review examines the literature on the use of automated (AI) methods for CAC scoring on non-ECG-gated chest CT scans, comparing them to the gold standard of Agatston CAC scoring. A potential gap in this literature that could be investigated is the use of automated (AI) methods for image noise reduction to improve non-ECG-gated CT scans, thereby increasing the sensitivity of this method for CAC scoring. It should be noted that similar methods of noise reduction for scoring have been investigated; however, the AI tool would perform the noise reduction only, and humans would perform the scoring [40].

Limitations of This Review

This scoping review has several limitations. By design, it does not include methodological quality or risk of bias assessments and therefore cannot be used to inform clinical guidelines [41-43]. Most included studies were small (<600 patients), with only two enrolling larger cohorts, and nearly all relied on retrospective datasets [21,26]. In addition, while some studies developed novel AI models for CAC scoring in non-ECG-gated CTs, many evaluated commercially available algorithms trained exclusively on ECG-gated CTs, which may limit accuracy [20-23,26]. Motion artifacts were frequently cited as a challenge, and scan comparisons were generally performed within a six-month window, rather than during the same session, which introduced potential bias due to disease progression and patient positioning. Because most datasets were retrospective convenience samples, selection bias and unmeasured confounding are likely. Limited adjustment for patient and acquisition factors (e.g., risk profile, heart rate, scanner/reconstruction parameters) may distort the apparent model performance.

Implications for Future Research

Considering the limits of this review, future studies should further explore the genre of AI-facilitated CAC scoring. This review was mainly limited to non-ECG-gated methods, while there is a need for more literature on ECG-gated CAC scores generated by AI. Moreover, nine of the 13 studies used the Agatston scoring system, the gold standard. The other four studies utilized other algorithms, and further research can be done on the compatibility of AI with different scoring algorithms and the resulting reliability and efficiency. Lastly, AI in the realm of cardiac risk stratification is a relatively new area of research. There is more to learn about the practicality of incorporating such technology into mainstream healthcare facilities.

## Conclusions

Coronary artery calcium (CAC) scoring is an important tool for cardiovascular risk stratification, yet current manual methods are limited by inefficiency. This scoping review demonstrates the potential of artificial intelligence (AI) to streamline non-ECG-gated CAC scoring, though further validation across diverse populations and settings is needed. Ethical and economic considerations must also guide implementation. These findings underscore AI’s promise not only for CAC scoring but for broader applications in cardiovascular care.
